# Social determinants of health in Canada: Are healthy living initiatives there yet? A policy analysis

**DOI:** 10.1186/1475-9276-11-41

**Published:** 2012-08-14

**Authors:** Dana Gore, Anita Kothari

**Affiliations:** 1 Faculty of Health Sciences Simon Fraser University, 8888 University Drive, Burnaby, BC V5A 1S6, Canada; 2 School of Health Sciences Faculty of Health Sciences, Western University, Arthur and Sonia Labatt Health Sciences Building, Room 222, London, ON, N6A 5B9, Canada

**Keywords:** Chronic disease prevention, Social determinants of health, Canada, Health policy, Healthy living, Policy analysis

## Abstract

**Introduction:**

Preventative strategies that focus on addressing the social determinants of health to improve healthy eating and physical activity have become an important strategy in British Columbia and Ontario for combating chronic diseases. What has not yet been examined is the extent to which healthy living initiatives implemented under these new policy frameworks successfully engage with and change the social determinants of health.

**Methods:**

Initiatives active between January 1, 2006 and September 1, 2011 were found using provincial policy documents, web searches, health organization and government websites, and databases of initiatives that attempted to influence to nutrition and physical activity in order to prevent chronic diseases or improve overall health. Initiatives were reviewed, analyzed and grouped using the descriptive codes: lifestyle-based, environment-based or structure-based. Initiatives were also classified according to the mechanism by which they were administered: as direct programs (e.g. directly delivered), blueprints (or frameworks to tailor developed programs), and building blocks (resources to develop programs).

**Results:**

60 initiatives were identified in Ontario and 61 were identified in British Columbia. In British Columbia, 11.5% of initiatives were structure-based. In Ontario, of 60 provincial initiatives identified, 15% were structure-based. Ontario had a higher proportion of direct interventions than British Columbia for all intervention types. However, in both provinces, as the intervention became more upstream and attempted to target the social determinants of health more directly, the level of direct support for the intervention lessened.

**Conclusions:**

The paucity of initiatives in British Columbia and Ontario that address healthy eating and active living through action on the social determinants of health is problematic. In the context of Canada's increasingly neoliberal political and economic policy, the public health sector may face significant barriers to addressing upstream determinants in a meaningful way. If public health cannot directly affect broader societal conditions, interventions should be focused around advocacy and education about the social determinants of health. It is necessary that health be seen for what it is: a political matter. As such, the health sector needs to take a more political approach in finding solutions for health inequities.

## Introduction

Preventative strategies focusing on healthy eating and physical activity, collectively known as healthy living, have become an important strategy in Canada for combating chronic diseases. Chronic diseases are rising to epidemic proportions in the Canadian population and costs associated with treating them pose a serious threat to the sustainability of the health care system [1]. Addressing the underlying causes of chronic diseases and their inequitable distribution through a preventative health promotion strategy has been acknowledged as an effective way to reverse these trends in both Ontario (ON) and British Columbia (BC). These provinces have recently reformulated their chronic disease prevention strategies as part of Canada's renewal of public health systems, initiated in 2003 as a response to Severe Acute Respiratory Syndrome (SARS). A common strategy that both provinces pursue is to address chronic disease prevention through healthy living initiatives - initiatives that work to promote healthy eating and physical activity as well as address other risk factors such as unhealthy alcohol consumption and tobacco use.

While healthy eating and physical activity were traditionally considered individual lifestyle choices, public health has shifted its perspective in the past several decades to encompass the broader context in which these choices are made. This includes daily living and working conditions that are not conducive to healthy lifestyles as well as broader structural determinants that create inequities between population groups, which together form the *social determinants of health*[2]. The World Health Organization (WHO) has defined the social determinants of health in the following way:

"The poor health of the poor, the social gradient in health within countries, and the marked health inequities between countries are caused by the unequal distribution of power, income, goods, and services, globally and nationally, the consequent unfairness in the immediate, visible circumstances of peoples lives – their access to health care, schools, and education, their conditions of work and leisure, their homes, communities, towns, or cities – and their chances of leading a flourishing life. Together, the structural determinants and conditions of daily life constitute the social determinants of health and are responsible for a major part of health inequities between and within countries" [2], p.1].

Within a Canadian context, some examples of social determinants of health that have been identified are: income and income distribution, education, unemployment and job security, employment and working conditions, early childhood development, food insecurity, housing, social exclusion, social safety net, health services, aboriginal status, gender, race and disability [3]. The social determinants of health have been consistently linked in the literature to chronic diseases such as cardiovascular disease, respiratory diseases, diabetes and cancer in Canada and worldwide; for example, it has been found that low socioeconomic status (SES), often measured by income and education levels, is associated with higher rates of cardiovascular disease, chronic obstructive pulmonary diseases, diabetes mellitus and asthma [4-9]. Research on Canadian cities has shown that people living in low income neighbourhoods experience significantly higher rates of chronic diseases such as diabetes and die several years earlier than their wealthier counterparts [10]. Job insecurity, characterized by unemployment, part-time employment and temporary employment, has been found to result in elevated blood pressure and higher risk of death from cardiovascular disease [11,12]. Even in a financially secure job, poor working conditions that place high demands on the worker, combined with low support and low job control have been correlated with elevated stress and increased rates of coronary heart diseases as well as higher risk of cardiovascular-specific mortality [13-15]. Nor are these trends colour-blind; racialized groups such as Aboriginal people, new immigrants and minorities of colour consistently earn lower incomes and experience higher rates of chronic disease than North Americans of European descent [16-19].

Canadian policy documents outlining priorities for public health have stressed the importance of an approach that addresses the social determinants of health [20-22]. In a recent high-level United Nations meeting on chronic diseases, the role that the social determinants of health play in chronic disease was recognized, as was the importance of addressing them in disease prevention strategies [23]. A delegation from Canada participated in this meeting, and Canada endorsed the resulting declaration [24]. An approach that effectively engages with the determinants has been suggested in Mikkonen and Raphael's *The Social Determinants of Health: The Canadian Facts*, and includes policies that offer a higher minimum wage, higher assistance levels for those unable to work, a more progressive taxation structure that redistributes income more fairly, increased unionization, better funding of public education, government regulation of post-secondary institution tuition, stronger legislation on anti-discrimination policies and equal opportunity hiring, a national childcare strategy, strategies to increase the affordability of nutritious food, increased spending on a housing strategy, policies that reduce barriers for refugees and immigrants to practice their professions, and recognizing Aboriginal government authority over a wider range of Aboriginal affairs [3].

Provincial health policy on chronic disease prevention for BC and ON corresponds to federal priorities. ON has stated in policy documents that the causes of chronic diseases are complex and rooted in broad determinants of health, which encompass lifestyle, socioeconomic, cultural and environmental factors [25,26]. In order to tackle these upstream causes, the province has decided on a comprehensive, integrated population health approach that is evidence-based [27,28]. For example, in a policy document on combating obesity, ON committed to a population health approach, which explores health disparities and interactions among the social determinants of health in order to improve the well-being of populations [26]. This approach can also be seen in the Ontario Action Plan for Healthy Eating and Active Living [28]. ON has made efforts to integrate a social determinants of health perspective into the province's public health practice through data and information use guidelines for boards of health of public health units. In the Healthy Eating, Physical Activity and Healthy Weights guidance document, the board of health is instructed to use information on health inequities and the social determinants in order to assess population needs and identify groups at highest risk [25].

BC shares fundamental similarities with ON in its agreement on the importance of the social determinants of health and the need for an evidence-based, population health approach to chronic disease prevention. In the Model Core Program Paper on chronic disease prevention in BC, the paper's working group identified key principles for successful disease prevention, which includes a focus on social, environmental and economic determinants of health, a "whole of society" approach to population health, and an equity lens to examine health disparities between different groups [29], p. iv]. The determinants of health are understood to interact with each other in a variety of ways, to compound vulnerabilities for certain sections of the population, and to be modifiable through health public policy and changing social norms. Like ON, BC also takes health disparities between different parts of the population into account, such as between men and women, different ethnic groups, those who identify as Aboriginal, or between people of varying socioeconomic status [29].

In order to effectively address the social determinants of health, ON and BC have committed to multi-sectoral action that targets the population on a variety of levels. Both BC and ON have stressed the importance of partnerships in the public health model to achieve prevention goals with other levels and sectors of government, NGOs, private industry, service providers, researchers, and communities to name a few [25,28,30-32]. These partnerships are considered crucial for real change, given the upstream, wide-ranging impact of the social determinants of health. Both provinces advocate for comprehensive strategies that target the population in diverse environments and at multiple levels [25,31].

These strategies suggest a socio-ecological approach to healthy eating and active living, a model of health that recognizes the interaction between individuals and their greater environment and its impact on health. In a socio-ecological model, the health behaviours that individuals engage in are impacted by individual factors (such as knowledge, lifestyle choices, and attitudes towards health behaviours), as well as interpersonal, community, organizational and society-level factors [33,34]. Interventions that follow this model aim to target the population at all these levels and address downstream as well as upstream barriers to healthy living [35]. It is important to note that although the socio-ecological model is different from the social determinants of health approach, it does not preclude attention to the social determinants of health. The social determinants of health can in theory be addressed at multiple levels within the socio-ecological framework, especially those that are more upstream such as at the societal level.

Based on the priorities that are listed above for healthy eating and active living in ON and BC, it would be expected that initiatives in both provinces target the population in diverse settings and at multiple levels, with a majority of initiatives focusing on improving population health through a social determinants of health framework. However, what has not yet been examined is the extent to which healthy living initiatives implemented under these new policy frameworks successfully engage with and change the social determinants of health.

This study seeks to evaluate healthy living initiatives in BC and ON that focus on healthy eating and physical activity based on their approach to the social determinants of health and health inequities. The authors draw on a political economy of health perspective in order to evaluate the initiatives within their broader social, political and economic context [36]. This implies that the findings related to the data are discussed in relation to the larger discourse around the socioeconomic environment and acknowledge the effect of structures and processes external to the health sector. Recommendations that emerge from the discussion are approached in a similar way.

## Methods

### Initiative search and selection strategy

Publically-available provincial policy documents written between 2005 and 2011 in BC and ON that focused on chronic disease prevention were used as a starting point to identify relevant initiatives. The focus was on chronic diseases that are most affected by nutrition and physical activity - namely cardiovascular diseases, some cancers, diabetes, hypertension, stroke and chronic respiratory disease. Once initiatives were identified, a web-based search was conducted to obtain a detailed description of the program as well as its timeline and current status (in-planning, ongoing, discontinued) etc. Provincial organizations that had the potential to conduct initiatives were also researched online to find initiatives that may not have been mentioned in the original policy documents and database. The search was not restricted to initiatives led by provincial ministries related to health; they could be initiatives of other ministries, arms-length government agencies or independent non-profit organizations that worked to improve healthy eating and physical activity. In order to ensure the list of healthy living initiatives was comprehensive, it was compared against recent documents and public databases that provide listings of healthy living initiatives at municipal, regional and provincial levels in BC and ON [26,37,38]. Lastly, two policy-makers in BC and ON reviewed the list of initiatives to fill any gaps.

### Inclusion criteria

a) Initiatives focused on healthy eating and physical activity as a preventative strategy to reduce chronic diseases or improve general health. The initiatives were not limited to government interventions - the initiatives could be funded and developed by various organizations including actors in the government, non-profit and private sectors.

b) Initiatives were implemented in BC and ON between January 1, 2006 and September 1, 2011.

c) Initiatives were provincial rather than regionally or nationally-based. Initiatives that focused on select sites in the province were also permitted provided they weren't restricted to a particular region or area. For example, an intervention targeting remote communities in ON would be acceptable for analysis, whereas an intervention led by a particular health authority and applied only to that health authority's region would not be included.

The findings are limited to the provincial scope of initiatives selected. It is difficult to determine if analysis of regional, municipal or community level initiatives would reveal convergent findings, and represents an area for future study.

### Analysis

Each of the identified initiatives was reviewed, analyzed and grouped using descriptive labels. Codes were developed directly from the data by asking the following questions of each initiative:

a) What section of the population does the initiative target? (ex. general public, vulnerable populations, health service providers, community actors, etc.)

b) What factor is the initiative trying to change that will lead to healthy eating and active living? (e.g. knowledge, skills, attitudes, built environment, access, social/economic/political factors, etc.)

c) Does the initiative directly acknowledge and attempt to act on the social determinants of health? If so, in what way? (e.g. education, advocacy, public policy change, etc.)

d) What is the mechanism that the initiative uses to promote healthy living? (e.g. direct program for population, resources, toolkits, consultation services, grants, etc.)

The predominant themes that emerged from questions b. and c. reflected three types of initiatives: lifestyle-based, environment-based, and structure-based, which were defined for this paper in the following ways:

• Lifestyle-based: These initiatives aim to improve healthy living through lifestyle change of individuals. Examples include raising awareness of the issues (e.g. obesity) in the general population, increasing knowledge around nutrition and physical activity, changing attitudes towards healthy living by appealing to social norms (e.g. social marketing campaigns) or directly encouraging the adoption of new behaviours through programs (ex. eating foods with lower salt content, exercising for 30 minutes each day). The target audience could be the general public or specific groups (e.g. low income individuals, children, or aboriginal people)

• Environment-based: These initiatives are meant to improve healthy living by influencing the immediate environment in which people spend their time, such as schools, workplaces and community spaces. Examples of these initiatives range from encouraging employers to initiate healthy workplace programs to banning the sale of unhealthy foods in schools or working towards a built environment that encourages physical activity. These initiatives were frequently settings-based and address the role that immediate environmental factors play in health.

• Structure-based: These initiatives directly acknowledge the impact of various structures (e.g. social, political, economic) that create inequities leading to chronic diseases and attempt to address the social determinants of health directly in order to improve healthy eating and active living. These types of interventions are most frequently centered around education and advocacy on the social determinants of health and worked specifically to correct health inequities caused by these structural conditions. Examples include a survey tool that assesses the cost of basic healthy eating in different geographic areas in order to monitor accessibility and affordability of a nutritious diet, the creation of community forums to discuss the social determinants of health and explore structural barriers to healthy living, or consultation services that provide gender equity audits to sport and recreation organizations. Of the three categories, this one is the only one that directly acts on the social determinants of health.

For each initiative type, it was also found using questions a. and d. that there was a broad variety of mechanisms by which the initiative was supported and delivered. Consequently, in each category, initiatives were classified according to the mechanism by which they were administered. Mechanisms were categorized as direct programs, blueprints, and building blocks, and were defined as the following:

• Direct program: Initiatives that are developed and implemented to directly influence the health of the population. Initiatives could be implemented through organization staff, contracting of other staff, working with community partners, or enforcing mandatory policy. Examples include direct services from health professionals such as phone lines staffed by dieticians and specialists in physical activity, bills to prohibit certain foods, and programs that provide healthy snacks to schoolchildren. This category also applies to programs that reward organizations (communities, schools) for programs they have already implemented.

• Blueprint: Initiatives that are developed but require implementation and tailoring by a third party such as a school, public health unit, or community organization. These initiatives are categorized as blueprints because while they offer a "plan" for a healthy eating and active living intervention (HEAL), they do not directly act on the population and their implementation is optional. Examples include toolkits for healthy school policies, materials for teachers to encourage student physical activity, and frameworks for how to build healthy communities. These initiatives require more action at the local level than direct programs because although the initiative is planned, local actors are needed to carry it through.

• Building Blocks: Initiatives that are meant to act as resources for third parties to develop their own projects, within certain guidelines. Examples include grants for communities to build their own HEAL project, consultation and training services on program planning, and directories of HEAL initiatives to act as a resource for ideas in developing an initiative. These initiatives require the most action at the local level; their planning and implementation fall to local actors and they provide the least support from the organization that is offering the initiative.

## Results

From the systematic scan of the policy documents, database and website search, 60 initiatives were identified in ON and 61 were identified in BC. (Please see Additional file 1 and Additional file 2 for a full list of initiatives). Programs were headed by various actors in both provinces, including Ministries of Health, other government sectors such as the Ministry of Education, non profit organizations, and professional associations. Often initiatives were structured as a partnership among multiple actors across different sectors. While many different organizations led and implemented healthy living initiatives, the majority were linked to provincial government in some way - either through direct funding, funding through an arms-length government agency (e.g. Cancer Care Ontario or Public Health Ontario), funding through a non-profit organization that has received sizeable grants for healthy living initiatives (e.g. BC Healthy Living Alliance), or partnership with a government agency. Government involvement in ON programming or financing included the Province of Ontario, the Ministry of Health and Long-Term Care, the Ministry of Child and Youth Services, the Ministry of Community and Social Services, the Ministry of Education, the (former) Ministry of Health Promotion and Sport, and the Ministry of Agriculture, Food and Rural Affairs. Government involvement in BC programming or financing included all Ministries, since all participated in ActNow BC. Some key ministries involved in healthy living initiatives were the Ministry of Health, the Ministry of Community, Sport and Cultural Development, the Ministry of Education, the Ministry of Agriculture and Lands, the Ministry of Children and Family Development, and the Ministry of Transportation and Infrastructure. Many initiatives involved multiple ministries and most included a health-related ministry. In ON, 6 of the 60 initiatives were not linked to the provincial government, and were organized and/or financed by Parks and Recreation Ontario, the Ontario Heart and Stroke Foundation, Dairy Farmers of Canada, and a partnership between the University of Guelph and the City of Guelph. In BC, 8 of the 61 initiatives were not linked to government, and were organized and/or financed by the BC Parks and Recreation Association, the Heart and Stroke Foundation of BC & Yukon, the BC Dairy Foundation, the Greater Vancouver Food Bank and Breakfast for Learning BC. For examples of initiatives classified into the three intervention types, please see Table 1. For examples of initiatives classified into the three delivery types, please see Table 2.

**Table 1:** Examples of healthy living initiatives in BC and ON according to intervention type: lifestyle-based, environment-based and structure-based

	**BC**	**ON**
**Lifestyle-based**	*Physical Activity Line*	*Healthy Babies, Healthy Children (HBHC)*
	A free telephone resource for British Columbians to receive information and advice from exercise physiologists on physical activity and healthy living.	Screening of children up to the age of 6 as well as parenting support, referrals and information on healthy practices such as breastfeeding, infant care and infant nutrition.
**Environment-based**	*Farm to School Salad Bar*	*Bill 8: Healthy Food for Healthy Schools Act*
	A program that connects schools with local farms in order to increase students' access to healthier food (e.g. fresh produce).	An amendment to ON's Education Act limiting the amount of transfats that can be sold on school property through means such as vending machines, special events and cafeterias.
**Structure-based**	*Everybody Active!*	*Nutritious Food Basket*
	A grants program for communities to begin a dialogue on how to address barriers to physical activity. It also provides resources on how social determinants of health such as poverty and social exclusion affect access to physical activity.	A survey tool that municipal boards of health are required to use in order to calculate the cost of nutritious food. This can be used to monitor how affordable and accessible foods are by comparing them to income levels of ON households

**Table 2:** Examples of healthy living initiatives in BC and ON according to mechanism of delivery: direct programs, blueprints and building blocks

	**BC**	**ON**
**Direct Program**	*Food Skills for Families*	*EatRight Ontario*
	A six week cooking program that is administered to 'at-risk' target populations. The program is administered by community facilitators, who have been trained by the BCHLA (the organization that offers this initiative).	Offers Ontarians free dietitian services on healthy eating and nutrition through a website, email, and toll-free number.
**Blueprint**	*LEAP BC*	*Teach Nutrition Programs and Resources*
	A set of written resources with activity ideas to help parents, caregivers and early learning practitioners encourage healthy eating and physical activity in young children.	A set of programs, written resources and workshops to help early childhood, elementary, and middle school teachers teach their students about nutrition and healthy eating.
**Building Blocks**	*Breakfast for Learning BC*	*HC Link*
	A program offering grants for start-up of community and school-based snack programs directed at children and youth and BC that include an educational component.	An organization that provides services to community organizations that aim to develop health promotion programs. Services include consultations, workshops and resources related to program planning, implementation, and evaluation.

In BC, 38 interventions were lifestyle-based, 27 were environment-based and seven were structure-based. Nine interventions had multiple components that targeted a combination of lifestyle, environmental and structural factors, and so were classified into more than one category. In terms of method of delivery, direct interventions were more prevalent in lifestyle-based initiatives: 18 initiatives used direct programming while 10 initiatives were blueprints and 10 were building blocks. In the environment-based category, there was more of a balance between mechanisms of delivery: a roughly equal number of environment-based initiatives worked through direct, blueprint and building block mechanisms (10, eight and nine, respectively). Structure-based interventions were those that received the least direct support: only one was enacted through direct programming, one used the blueprint format, and six were building blocks-type initiatives.

ON yielded similar results in terms of distribution - the preponderance of initiatives were lifestyle-based, followed by environment-based, with very few aimed at structural change. Of 60 provincial initiatives identified, 36 were lifestyle-based, 26 were environment-based and nine were structure-based. Six interventions had multiple components that targeted a combination of lifestyle, environmental and structural factors, and so were classified into more than one category. Most lifestyle-based interventions were direct (23), while 11 were blueprint initiatives and four were building blocks. Environment-based initiatives were also more likely to be direct (13) while nine were blueprints and four were building blocks. Of initiatives that acted at a structural level, two acted through direct mechanisms, three were blueprints and four were building blocks.

In summary, BC and ON had similar distributions of intervention types, with the majority falling into lifestyle-based initiatives, followed by environment-based initiatives, and a small proportion falling into the structure-based category. While many initiatives focused on changing lifestyle and the immediate environment to improve healthy eating and physical activity, very few were directed towards changing more upstream social determinants of health, such as the economic and social conditions that create inequities between genders, income groups and ethnic groups. Only 11.5% of initiatives in BC and 15.0% of initiatives in ON had structural components that directly spoke to the social determinants of health.

**Figure 1:**
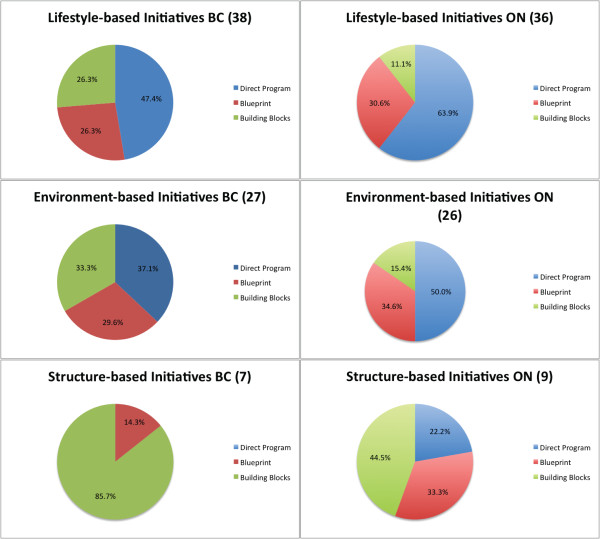
**Mechanisms of initiative implementation for lifestyle-based, environment-based and structure-based initiatives in BC and ON between January 1, 2006 and September 1, 2011.** Mechanisms of initiative implemented included direct programs, blueprints, and building blocks and were calculated for a total of 61 initiatives in BC and 60 in Ontario. Please note that percentages may not add to 100% because some initiatives operated by more than one mechanism and so were placed in multiple categories.

In terms of the mechanism by which the intervention was implemented, ON had a higher proportion of direct interventions than BC for all intervention types (63.9% vs. 47.4% for lifestyle-based interventions, 50.0% vs. 37.1% for environment-based interventions, and 22.2% vs. 14.3% for structure-based interventions). However, the same trend can be observed for both provinces: as the intervention becomes more upstream and attempts to target the social determinants of health more directly, the level of direct support for the intervention lessens. In BC direct programming drops from 47.4% for lifestyle-based initiatives to 37.1% for environment-based initiatives to 0% for structure-based initiatives. In ON direct programming drops from 63.9% for lifestyle-based initiatives to 50.0% for environment-based initiatives to 22.2% for structure-based initiatives. For a visual representation of this trend, please see Figure 1.

## Discussion

### The problem at hand

The dominance of lifestyle-based and environment-based initiatives is troubling considering that initiatives were expected (and directed) to focus on the social determinants of health. Although individual behaviour change theories were popular early in the health promotion movement, the field of public health has matured to embrace a more multi-level approach. This change of focus was in recognition of the fact that individual behaviour change strategies are not enough for lasting health improvements, given structural conditions which predispose people to illness [39-41]. They may actually be counterproductive; they tend to place responsibility to change directly on individuals and can lead to victim-blaming should barriers prove too great for them to be successful [42-44]. The individual change strategy can be particularly problematic when it comes to addressing the impact of inequities on vulnerable populations, considering that such interventions often focus on increasing knowledge, changing attitudes and/or encouraging adoption of healthy behaviours. This approach in a marginalized group runs the risk of implying that the group is to blame for their higher rates of chronic disease, purportedly due to their own ignorance of healthy living or lifestyle choices.

Environment-based interventions, while more sensitive to the context in which people live and work, still do not tackle the structural determinants which create these conditions in the first place. Programs aiming to increase access to nutritious food and physical activity in particular settings such as schools, workplaces, government buildings and communities do not alter the factors which create inequities and unfavourable living conditions [45]. Environment-based initiatives can also have potentially negative implications for health equity when applied at a population level with no consideration for differential access. Programs that "treat everyone the same" and fail to acknowledge different positions vis-à-vis the social determinants of health may in fact benefit those who already have access while excluding those who are more vulnerable. This effect was succinctly illustrated in Frohlich and Potvin's critique of Geoffrey Rose's population strategy [46].

What is needed are structural interventions that are inherently redistributive in nature; interventions that broaden the distribution of power, income, goods and services across the population. Research has demonstrated limited effectiveness of downstream interventions (such as programs that focus on behaviour change) when structural barriers are not addressed [47-50]. This is not surprising, considering that structural determinants have been found to influence the distribution of risk factors for chronic diseases such as smoking behaviour, overweight and obesity, and physical inactivity [8,51,52]. Therefore, attempting to prevent chronic disease by targeting risk factors at the individual or environmental level may not be effective without also addressing the broader determinants that shape those risk factors.

As can be seen from the paucity of structural interventions, this approach to healthy living is lacking in ON and BC despite provincial policy direction. Another discouraging trend found in the data is the decrease in direct initiatives (direct programs) and increase in more indirect ones (at the blueprint and building blocks levels) as the initiatives become more upstream. The amount of indirect initiatives could be interpreted as a move towards increasing community capacity, inclusion, local responsiveness and decision-making in healthy living initiatives. However, the more upstream and broad-scale an intervention is (i.e. an environment or structure-based program), the more it would benefit from coordinated action at a higher, more structural level [3]. Initiatives that address the social determinants of health in order to impact the population at a broad level can extend beyond the scope of a particular community organization, whose on-going population reach and resource availability are limited.

Health equity interventions can also require more direct action from government - for example, an initiative that would help to balance the distribution of wealth in Canada is a more progressive taxation structure or an increase in minimum wage to account for inflation and provide a higher standard of living. These are initiatives which cannot be undertaken by individuals and communities. As the word 'structural' implies, they need to be acting directly on the structures (economic, social, political, etc.) which create and maintain health inequities. Instead, communities that do not have that capacity are more likely to receive this responsibility - in the form of grants, training workshops for program planning, or resource directories. These initiatives are framed as supporting communities in building their own initiative that addresses the social determinants of health. However, the pressure that it places on communities is enormous, and allows the public health system to abdicate its responsibility to address the social determinants of health directly and in a concrete manner. Instead it can point to these initiatives and claim that they are focussing on the social determinants of health - this is essentially the provinces of ON and BC "passing the buck".

### Context for the contradiction

Given the fact that policy documents on chronic disease prevention and healthy living at the provincial level in both BC and ON acknowledge the importance of the social determinants of health, why is the health sector not acting on them? An explanation can be found in the context of Canada's political and economic policy over the past several decades. A neoliberal approach to the economy that favours freedom of the market has resulted in the retraction of government intervention in the areas that are crucial to the health and well-being of Canadians. Research has found that Canadians are experiencing increasing levels of poverty and income inequality, as absolute levels of poverty increase and the gap between the poorest 10% and richest 10% widens [53]. Research based in Toronto has demonstrated this trend of polarization starting from the 1970s [54]. Urban poverty is also becoming more concentrated in peripheral areas - areas that have the highest rates of new immigrants and visible minorities. Not surprisingly, child poverty in Canada has also deepened in the 1990s [55].

Other determinants of health have also been affected by government policy. The public education system has suffered cutbacks and labour conflicts that reduce its ability to provide quality education [56]. Stricter immigration policy that went into effect as of December 2011 will increase social exclusion of immigrants and refugees, while cutbacks to legal aid aggravate the situation [57]. Job insecurity is rising, with the percentage of people in full-time jobs decreasing and the number of people working part-time, in shift work, temporary contracts or self employed increasing [58]. Unionization rates have also dropped across the country [59]. Disproportionate spending on necessities such as housing comes hand in hand with increasing poverty and job insecurity. Canada is experiencing a national housing and homelessness crisis. As of 2007, over 35% of people renting in major urban areas such as Toronto, Montreal and Vancouver were spending more than 30% of their income on housing (the cut-off for affordable housing). Around 20% are spending more than 50% of their income on rent, which puts them at risk of homelessness [53]. When such a high amount of income is being devoted to shelter, not enough is left over for nutritious food, leading to food insecurity [60].

The effects are felt as a result of inadequate policy and public expenditures on social programs, which are key characteristics of the neoliberal model. Public spending on family-related benefits has been scaled back since the 1980s, and taxation policy between 1990 and 2005 has increased the tax burden on the bottom 10% of income earners and relieved it from the top 1% [53]. Minimum wage, although it has increased in absolute terms, has fallen behind the inflation rate and made living above the poverty line more difficult to achieve. The fall of unionization in BC and ON can be attributed to policies put into place by Conservative governments that made unionization more difficult [53]. With respect to housing policy, it has been argued that a budgetary increase of 1% in Canadian government spending at the federal, provincial, territorial and municipal levels has the potential to end the homelessness crisis, but they have demonstrated their unwillingness to make that commitment [61].

Within the context of a national and provincial neoliberal climate, it is not surprising that the health sectors of BC and ON have not attempted to implement widespread structural change to improve healthy living [62-64]. Even though well aware of the necessity to address the social determinants of health, they may feel powerless to do so in the face of conservative policies initiated by other sectors. As Alvaro et al. emphasized using a critical theory lens, government departments linked to economics and ensuring the dominance of the free market have more power than departments such as the Ministry of Health in a neoliberal model [45]. Those in the health sector face barriers to encouraging other sectors to effect policy change to improve the social determinants of health, and may resort to individual or intermediate behaviour change because they are able to effect that change either through their own department or allied with other de-prioritized departments such as the Ministry of Education or the Ministry of Environment. For example, partnering with schools to increase the amount of healthy foods sold in vending machines may be significantly easier than convincing the Department of Finance to raise the province's minimum wage.

### The way forward

We would argue that the ultimate goal of healthy living programs should be to improve the social determinants of health and eliminate health inequities. It is recognized that it is out of the scope of the health care sector to effect those changes on its own, and it faces barriers in partnering with sectors for collaborative, cross-sectoral action. However, public health should be constantly attempting to move towards those goals. It should not settle for programs that bring about changes in lifestyle and the immediate environment while only addressing the social determinants model at a conceptual level.

If programs cannot directly affect lasting, broader societal conditions, interventions should be focused around advocacy and education about the social determinants of health - advocacy at the level of the population, service providers, health organizations, and government in order to build political will to address them. The structural interventions listed in Additional file 1 and Additional file 2 are already taking the initiative to do this and more should be added.

One barrier for public health professionals to address the social determinants of health is a lack of understanding of how to do so; although there is a wealth of theoretical understanding of how these determinants affect health, there have been few examples to date that illustrate how to effectively change them [62,65]. In an environmental scan of the integration of the social determinants of health with public health practice, the National Collaborating Centre for Determinants of Health noted that implementation of programs that dealt with the social determinants of health in Canada was relatively scarce and, when extant, in early phases [66]. Some of the barriers noted to mounting programs that focused on social determinants included gaps in the existing evidence base on the social determinants of health and on interventions that were effective in addressing them, difficulties public health professionals faced in conceptually differentiating individual-level and population-level approaches, a lack of clarity on where in the path from determinants to outcomes public health is expected to act, and limitations in current public health practice methods, which rely mostly on quantitative data.

Even in a conservative political climate, it is clear that there are improvements that can be made within public health to foster a greater understanding of how to focus programming on the social determinants of health. The WHO Commission on the Social Determinants of Health notes that a comprehensive health equity surveillance system would capture the most upstream structural drivers of health inequities (the unequal distribution of power, money, goods and services) as well as more intermediate ones that encompass the daily conditions in which people live and work. Such a system could monitor health equity by stratifying morbidity and mortality data by indicators such as income, occupation, gender, region, ethnicity and immigration status [2]. Some such initiatives already exist, for example the EU Health Monitoring Programme, which could be used as a model for Canada [67]. Solid data on health inequities and the social determinants of health serve a dual purpose: not only do they allow public health professionals and provincial health care systems to understand inequities and design effective initiatives that address structural determinants, they can also be used as tools to advocate for change at a broader level, which may be outside the scope of the public health system. For example, data on the health effects of social exclusion faced by new immigrants and refugees could be used to advocate for progressive immigration policies.

It is equally important that health organizations and professionals know how to use evidence on inequities and the social determinants of health to create meaningful initiatives. To do this, there must be a comprehensive understanding among the healthcare force of the social determinants of health and how they affect populations. This includes awareness of the social, political and historical context of how these inequities are generated and continue to be maintained. The provincial health services authority in BC has a program modeling this principle called the *Indigenous Cultural Competency Online Training Program*[68]. This program consists of a series of online modules and discussions designed to educate health professionals across the province on the context surrounding Aboriginal health issues, including the history of colonization in BC, Indian residential schools and hospitals, structural and interpersonal racism, and their impacts on Aboriginal peoples and their health. It would be extremely useful to have such programs implemented in all provinces, ideally with specific sections that focus on chronic disease, as rates of chronic diseases such as diabetes and cardiovascular disease are much higher in Aboriginal populations.

With solid evidence and a comprehensive understanding of inequities, there are many ways that public health can begin to address the social determinants of health in programming. One possibility is using public health planning models that integrate the social determinants of health into the planning process. The Region of Waterloo Public Health in ON developed a planning model that does this, based on the Ontario Public Health Standards (OPHS) [69]. The model is called *Evidence and Practice-based Planning Framework: with a focus on health inequities*. In the first two steps of program planning (1. Define Issue, 2. Situational Assessment), planners are encouraged to consider the following: community health needs, the OPHS mandate on the social determinants of health, and the association between health status and the determinants of health. Further, they are asked to engage stakeholder perspectives [69]. Another model developed by the National Public Health Partnership in Australia makes the determinants of health even more central to the planning process [70]. This framework bases the intervention on the determinant that is causing the health problem, rather than the health problem itself. Public health teams are to identify the determinants of the health problem and their context, assess how determinants may be detrimental or protective, appraise different intervention options, decide on an option - taking into consideration its impact on health equity, then implement and review it [70]. When consistently implemented province-wide these types of planning models will help public health teams incorporate the equity and the social determinants of health into practice in a systematic manner.

Information and programs generated within the public health sector can be used to advocate for structural change to improve healthy living. An exemplary initiative in Ontario is the *Nutritious Food Basket*, described in Table 1. The *Nutritious Food Basket* is a program mandated by Ontario Public Health Standards (OPHS) for boards of health to implement in municipalities across the province. Boards of health are required to survey local supermarkets and grocery stores in order to calculate the cost of basic healthy eating for individuals and families. This program is ideal for a number of reasons. It links what is normally considered a behaviour (healthy eating) to greater structural determinants such as income and regional differences in food accessibility. Because the survey is taken annually, it can keep pace with larger economic trends such as inflation and food cost patterns, and because it is performed systematically using a detailed protocol it presents reliable data. The data, as mentioned in the Nutritious Food Basket Protocol, can be used for program planning, policy decisions, and advocating for accessible, affordable foods. The *Nutritious Food Basket* can be used as powerful evidence for the necessity of income redistribution policies ensuring that families make enough money to maintain a healthy diet [71]. Certain boards of health, for example in the Cities of Hamilton and Sudbury, have used this tool for this purpose [72-74].

A current leader in championing health inequities is the Sudbury & District Health Unit, whose team has launched public awareness campaigns linking the social determinants to health outcomes, created health planning and mapping tools that focus on equity, established in conjunction with the City of Sudbury a Food Charter that recognizes food as a basic human right, and developed a primer for municipal leaders explaining the connections of social determinants to public health and how they could address them effectively [75]. Although individual public health units are to be commended for their leadership, coordinated action at the provincial level would be much more influential.

External evidence from other countries can also be used as leverage - for example healthy living and chronic disease policy in Northern European countries such as Sweden and Norway. Sweden initiated a public health policy in 2000 which stressed improving employment conditions and decreasing poverty as primary goals for improving health [76]. Sweden has significantly lower obesity rates than Canada and research has shown obesity trends levelling off between 2000/2001 and 2004/2005 [77]. Elizabeth Fosse has pointed out that Norway focuses on structural measures that function to redistribute resources within society, which is characteristic of a social democratic welfare state [78]. In a 2005 health policy document, the Norwegian government outlined a number of strategies to combat health inequities, including reducing inequalities that contribute to poor health [78]. The government pledged to work to provide safe childhood conditions, fair income distribution, and equal opportunities in work and education. It was also recognized by the Norwegian government that individual behavioural choices which impact healthy living are influenced by broader structural determinants, and therefore the government must work to address those determinants by influencing cost and availability of resources to healthy living [78]. Lastly, a strategy employed to reduce inequities was to develop all initiatives to maximize social inclusion of all citizens. These types of policies could be used as models for health inequity reduction strategies advocated by the health sector in BC and ON.

This study is not without limitations. For example, the focus on provincial-level initiatives excluded initiatives happening at regional, municipal and community levels. This selection was strategic in that it attempted to maximize the likelihood of finding initiatives which addressed the social determinants of health - conditions that require multi-sector, systemic change. It was assumed that this type of change more likely to happen at the provincial level as opposed to in a city or region, but it is possible that initiatives that address the social determinants of health at a more local level were overlooked. Secondly, our search strategy was limited to initiatives that focused explicitly on healthy eating and active living and did not seek to identify social programs in other sectors (for example housing) that may address the social determinants of health and impact healthy eating and active living indirectly. We would like to emphasize, however, that our focus was on what is occurring within public health at a provincial level to improve healthy eating and active living. The presence of social programs in other sectors does not reduce public health's obligation or commitment to addressing the social determinants of health. Finally, our analysis did not attempt to document whether desired outcomes related to the social determinants of health were achieved by the searched initiatives; such outcomes require many years to manifest themselves.

## Conclusions

Addressing the social determinants of health necessarily means moving away from depoliticized frameworks that emphasize biomedical factors in disease. Attention to the social determinants and inequities has been growing, as health promotion movements evolve - movements that were initially led by Canada. However it is necessary that health be seen for what it is: a political matter. As such, the health sector needs to diversify to a more political approach in finding solutions for health inequities. Until this occurs, it is debatable how much progress can occur on improving the social determinants of health.

## Competing interests

The authors declare that they have no competing interests.

## Authors' contributions

DG conceived of the study, lead the analysis and drafted the manuscript. AK participated in refining the study's design, acted as a critical discussant of analytical findings and helped to draft the manuscript. Both DG and AK read and approved the final manuscript.

## Supplementary Material

Additional file 1 BC Healthy eating and active living initiatives analyzed (61). Click here for file

Additional file 2 ON Healthy eating and active living initiatives analyzed (60). Click here for file
