# Supplementary material for: Social determinants of health in Canada: Are healthy living initiatives there yet? A policy analysis
Source: Int J Equity Health. 2012 Aug 14;11:41. doi: 10.1186/1475-9276-11-41 (PMC3492195; doi:10.1186/1475-9276-11-41)
Supplement: Additional file 1 — BC Healthy eating and active living initiatives analyzed (61). [file 1475-9276-11-41-S1.pdf]

Additional File 1: BC Healthy eating and active living initiatives analyzed (61)

	Lifestyle-based (38)	Environment-baesd (27)	Structure-based (7)
**Direct (28)**	1. Farm to School Salad Bar*<br>2. Food Skills for Families<br>3. Sip Smart BC<br>4. ActNowBC Public Awareness Campaign<br>5. ActNowBC Daily Physical Activity Program*<br>6. Dietitian Services at Healthlink BC<br>7. Healthy Families Walking Challenge<br>8. Prescription for Health<br>9. Healthy Families BC Informed Dining Program<br>10. Public Awareness Campaign around Sodium/sweetened Beverage Reduction<br>11. Physical Activity Line<br>12. Patient Voices Network Peer Coaching Program<br>13. Active Choices-Self Management Program<br>14. Healthy Buddies<br>15. Shapedown BC<br>16. Boomer Friendly Boot Camp<br>17. Hearts in Motion Walking Club<br>18. Power of Sport Tour	1. Farm to school salad bar*<br>2. BC School Fruit and Vegetable Nutritional Program<br>3. ActNow BC Daily Physical Activity Program*<br>4. Guidelines for Food and Beverage Sales in BC Schools<br>5. Transfat Policy under the Public Health Act<br>6. Produce Availability in Remote Communities Initiative<br>7. BC Seniors Community Parks<br>8. Healthy Convenience Store Initiative<br>9. BC Healthy School Network<br>10. Bike BC	0
**Blueprints (19)**	1. Walk BC<br>2. Hub for Action on School Transportation Emissions<br>3. HeartSmart Kids<br>4. Action Schools BC*<br>5. International Walk to School Week<br>6. FreshChoice Kitchens*<br>7. Healthy Eating and Physical Activity Learning Resource<br>8. Nutrition Education Workshops and Resources<br>9. Literacy, Education, Activity and Play (LEAP) BC<br>10. Move for Health day	1. School Guidelines Support<br>2. Stay Active Eat Healthy<br>3. Healthy Work Environments<br>4. Action Schools BC*<br>5. Healthy Fundraising for Schools<br>6. FreshChoice Kitchens*<br>7. School Milk BC<br>8. Healthy Living Schools Initiative	1. Promotion Plus Consultation Services
**Building Blocks (25)**	1. Community-based Awareness<br>2. Active Communities*<br>3. Breakfast for Learning BC*<br>4. Community Health Promotion Fund*<br>5. Community Food Systems for Healthy Living*<br>6. Success by 6*<br>7. Aboriginal Seed Grant Program<br>8. Girls Only Incentive Program<br>9. Fab 55+ Incentive Program<br>10. Kidsport BC	1. Build Environment & Active Transportation (BEAT) BC<br>2. Active Communities*<br>3. Breakfast for Learning BC*<br>4. Community Health Promotion Fund*<br>5. CommunityLINK fund<br>6. Community Food Systems for Healthy Living*<br>7. Success by 6*<br>8. Seed Grant Communities<br>9. Local Motion	1. Everybody Active<br>2. Community Food Action Initiative<br>3. BC Healthy Communities<br>4. Aboriginal Health Initiative Program<br>5. Community Health Promotion Fund*<br>6. Community Food Systems for Healthy Living

*Initiatives have components that fit into multiple categories such as lifestyle-based and environment-based, or blueprint and building blocks.
