# Supplementary material for: Social determinants of health in Canada: Are healthy living initiatives there yet? A policy analysis
Source: Int J Equity Health. 2012 Aug 14;11:41. doi: 10.1186/1475-9276-11-41 (PMC3492195; doi:10.1186/1475-9276-11-41)
Supplement: Additional file 2 — ON Healthy eating and active living initiatives analyzed (60). [file 1475-9276-11-41-S2.pdf]

Additional File 2: ON Healthy eating and active living initiatives analyzed (60)

	Lifestyle-based (36)	Environment-based (26)	Structure-based (9)
**Direct (38)**	1. Eat Right Ontario<br>2. Healthy Babies, Healthy Children<br>3. Have a Ball<br>4. Community Food Advisor Program<br>5. Colour It Up<br>6. Fuel Up for Fun<br>7. Busy Bodies<br>8. Eat Right Be Active<br>9. Nutristep<br>10. Healthy Measures*<br>11. PlaySport<br>12. Ontario's After-school Program<br>13. Active2010 website<br>14. Healthy Ontario website<br>15. Physical Activity and Sport for Women Aged 55 to 70+ Program<br>16. Vegetable and Fruit Radio Ads<br>17. Prends-toi en Main!<br>18. Project ACT/Projet ACTIF<br>19. Daily Physical Activity<br>20. Everyone Jump<br>21. Back to Nature<br>22. Heart and Stroke Foundation of Ontario website<br>23. Walk This Way	1. Bill 8: Healthy Foods, Healthy Schools<br>2. Healthy Schools Recognition Program<br>3. Eat Smart<br>4. Healthy Food Guidelines for the Great Waterfront Trail Adventure<br>5. Marketing to Children Workgroup<br>6. Mars Landing<br>7. Ontario Trails Strategy<br>8. Trails Open Ontario<br>9. Future of the Good Food Box Program<br>10. Healthy Communities and the Built Environment<br>11. Daily Physical Activity<br>12. Back to Nature<br>13. Youth Friendly Community Recognition Program	1. OCDPA Advocacy Work (ex. common messages)<br>2. Nutritious Food Basket
**Blueprint (23)**	1. Ontario Heart Health Program Policy Framework*<br>2. Minding our Bodies*<br>3. Let's be Healthy Together*<br>4. Healthy Measures*<br>5. Living Schools<br>6. Health and Physical Activity Support Documents<br>7. Obesity Messages: Making them Stick<br>8. OPHEA Early Learning Resource<br>9. Vibrant Faces<br>10. Ontario Agri-Food Education (OAFE) Teaching Resources<br>11. Teach Nutrition Programs and Resources	1. Ontario Heart Health Program Policy Framework*<br>2. Minding our Bodies*<br>3. Online Activity Planner<br>4. Health and Physical Activity Curriculum Support Documents<br>5. Comprehensive Workplace Health Promotion (CWHP) Project<br>6. Ontario Student Nutrition Program<br>7. Project ACT/Projet ACTIF<br>8. OPHEA Daily Physical Activity Supports<br>9. Inclusion Resources (OPHEA)	1. Healthy Communities: An Approach to Action on Health Determinants in Canada<br>2. Creating Inclusive Community Organizations<br>3. Let's be Healthy Together (toolkit for service providers)*
**Building Blocks (12)**	1. Spark Together for Healthy Kids*<br>2. Healthy Communities Fund*<br>3. HC Link*<br>4. Physical Activity Resource Centre	1. Spark Together for Healthy Kids*<br>2. The Children's Collaborative<br>3. Healthy Communities Fund*<br>4. HC Link*	1. Spark Together for Healthy Kids*<br>2. Health Promotion Hub<br>3. FoodNet Ontario Database<br>4. HC Link*

*Initiatives have components that fit into multiple categories such as lifestyle-based and environment-based, or blueprint and building blocks.
